# The tomato histone deacetylase *SlHDA1* contributes to the repression of fruit ripening and carotenoid accumulation

**DOI:** 10.1038/s41598-017-08512-x

**Published:** 2017-08-11

**Authors:** Jun-E Guo, Zongli Hu, Mingku Zhu, Fenfen Li, Zhiguo Zhu, Yu Lu, Guoping Chen

**Affiliations:** Laboratory of molecular biology of tomato (Chongqing University), Ministry of Education, Bioengineering College, Chongqing University, Chongqing, 400044 People’s Republic of China

## Abstract

Histone deacetylation is one of the well characterized post-translational modifications related to transcriptional repression in eukaryotes. The process of histone deacetylation is achieved by histone deacetylases (HDACs). Over the last decade, substantial advances in our understanding of the mechanism of fruit ripening have been achieved, but the role of *HDACs* in this process has not been elucidated. In our study, an RNA interference (RNAi) expression vector targeting *SlHDA1* was constructed and transformed into tomato plants. Shorter fruit ripening time and decreased storability were observed in *SlHDA1* RNAi lines. The accumulation of carotenoid was increased through an alteration of the carotenoid pathway flux. Ethylene content, ethylene biosynthesis genes (*ACS2*, *ACS4* and *ACO1*, *ACO3*) and ripening-associated genes (*RIN*, *E4*, *E8*, *Cnr*, *TAGL1*, *PG*, *Pti4* and *LOXB*) were significantly up-regulated in *SlHDA1* RNAi lines. In addition, the expression of fruit cell wall metabolism genes (*HEX*, *MAN*, *TBG4*, *XTH5* and *XYL*) was enhanced compared with wild type. Furthermore, *SlHDA1* RNAi seedlings displayed shorter hypocotyls and were more sensitive to ACC (1-aminocyclopropane-1-carboxylate) than the wild type. The results of our study indicate that *SlHDA1* functions as a negative regulator of fruit ripening by affecting ethylene synthesis and carotenoid accumulation.

## Introduction

Fruit ripening is a complex regulated process that involves numerous metabolic changes, such as changes in color, flavor, aroma and nutrition. The process is controlled by endogenous hormonal^1, 2^ as well as genetic regulators and external signals (temperature, light and hydration)^3^. Ripening allows fruit to facilitate seed dispersal and provides essential nutrition in the human diet. In climacteric fruits (e.g. tomato, apple and banana), ethylene plays important roles in fruit development and ripening and is an essential factor for the ripening process^4, 5^. Respiration is dramatically induced and the ripening of fruit is initiated by ethylene biosynthesis in climacteric fruits^6^, which is different from the case in non-climacteric fruits (e.g. grape and citrus). There are two key biosynthetic enzymes in the ethylene biosynthesis pathway: ACS (1-aminocyclopropane-1-carboxylate synthase), which transforms SAM (*s*-adenosyl-*l*-methionine) to ACC (aminocyclopropane-1-carboxylic acid)^7^ and ACO (1-AMINOCYCLOPROPANE-1-CARBOXYLATE OXIDASE), which converts ACC to ethylene^8^. In *SlACS2* RNAi transgenic tomato fruits, ethylene production and fruit ripening are obviously inhibited^8^. Previous studies also revealed that RNAi inhibition of *SlACO1* delays ripening of climacteric fruits^9, 10^. These findings indicated that normal function of ethylene biosynthesis is essential for the ripening process.

In addition to ethylene synthesis, the ability to perception and response to ethylene is necessary for fruit ripening. The expression of *E4* in fruit is rapidly up-regulated following exogenous ethylene induction^11^. In fruit, *E4* transcripts are suppressed by ethylene biosynthesis inhibition^12^. *E8* is another a ripening-associated, fruit-specific expression gene in tomato that is regulated by ethylene^13^. Thus, illuminating regulation of these gene activities is important for us to understand the processes of ripening.

Tomato is usually considered to be an excellent model plant for studying climacteric fruit ripening. To date, the regulatory mechanisms controlling fruit ripening in tomato have been studied extensively. In these studies, a series of natural ripening-deficient mutants in tomato, such as *rin*, *Nr*, *Cnr* and *TAGL1* have facilitated our understanding of the transcriptional control system underlying tomato ripening^14–19^. For example, the *rin* mutant displays inhibited fruit ripening and enlarged sepals, which have phenotypes ascribed to the function of two MADS-box transcriptional factors, *SlMADS-RIN* and *SlMADS-MC*. *SlMADS-RIN* regulates fruit ripening, and *SlMADS-MC* is involved in sepal development and the formation of abscission zones^18^.

Histone acetylation is often associated with activation of transcription, whereas histone deacetylation is correlated with transcriptional repression. Histone acetylation levels are determined by the action of HATs (histone acetyltransferases) and HDACs (histone deacetylases). Over the past decades, an increasing number of HDACs have been identified in plants. There are 18 *HDAC* genes in *Arabidopsis*, 18 *HDAC* genes in rice, 5 *HDAC* genes in maize^20^ and 14 *HDAC* genes in tomato^21^. *HDACs* have been grouped into subfamilies: RPD3/HDA1, HDT and SIR2^22, 23^. In tomatoes, nine HDACs belong to the RPD3/HDA1 subfamily(SlHDA1–9)^24^, three belong to the HDT subfamily(SlHDT1, SlHDT2 and SlHDT3) and two belong to the SIR2 subfamily(SIR1 and SIR2). Based on domain organization and phylogenetic relationships the RPD3/HDA1 subfamily was subdivided into four groups: class I (SlHDA1, SlHDA2, SlHDA3 and SlHDA4), class II (SlHDA7, SlHDA8 and SlHDA9), class III (SlHDA5) and class IV (SlHDA6). Until now, there have been some reports on *HDACs* in *Arabidopsis* and rice, but rarely in tomato.

Here, we report the functional characterization of a *HDAC* gene, *SlHDA1*, isolated from tomato fruits based on a cDNA clone. A previous report indicated *SlHDA1* is mainly expressed in fruit and its transcript increases along with fruit development and ripening^21^. However, to date, *SlHDA1* has not been studied for its functional attributes in tomato. In this study, RNAi repression of *SlHDA1* was performed to investigate the exact role of *SlHDA1* in tomato, and the results conformed our supposition that *SlHDA1* acts as an inhibitor of fruit ripening.

## Results

### Creation of *SlHDA1* RNAi lines

**Figure 1:**
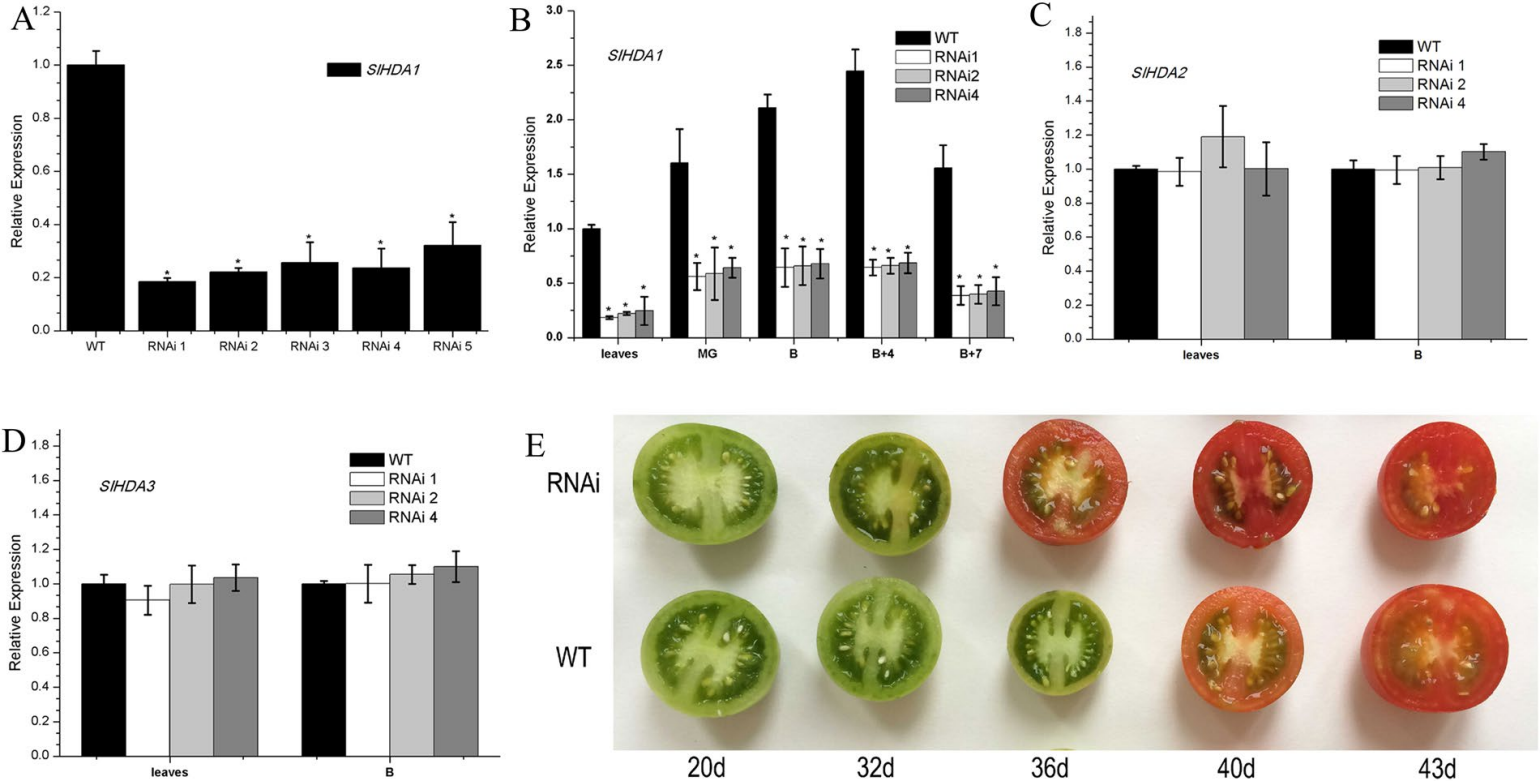
Phenotypic and gene expression analyses of *SlHDA1* in RNAi lines. (**A**) Expression of *SlHDA1* in RNAi lines and wild type (WT). RNAs were extracted for qPCR assay from leaves of RNAi lines and the wild type. (**B**) Relative expression profiles of *SlHDA1* between WT and *SlHDA1* RNAi lines. The WT expression data in leaves are normalized to 1. (**C**) and (**D**) Other two *SlHDAC* genes expression in *SlHDA1* RNAi lines and wild type fruits. (**E**) Fruits phenotype of wild type and *SlHDA1* RNAi lines. 20d-43d, statistical time starting from the pollination. *SlHDA1* RNAi lines changed earlier 3–6 days than wild type. 15–20 fruits were examined for biological replicates per line. Three biological replications and three technical replications for each sample were performed. Data are the means ± SD of three independent experiments. The asterisks indicate statistically significant differences between the WT and transgenic fruits (P < 0.05).

To gain further insight into the function of the *SlHDA1* gene, five independent *SlHDA1* silenced lines were obtained using RNAi. Total RNA was isolated from leaves, MG, B, B + 4 and B + 7 stage fruits of transgenic and wild type tomatoes. Real-time quantitative PCR (qPCR) results showed that the relative expression of *SlHDA1* was significantly reduced in five transgenic lines compared with the wild type (Fig. 1A). Three independent transgenic lines (lines 1, 2 and 4) exhibiting distinguishable alterations were selected for further characterization. As shown in Fig. 1B, the transcripts of *SlHDA1* were significantly reduced to approximately 20–30% of the control levels in RNAi lines in all detected tissues. Notably, in wild type, the *SlHDA1* gene was highly expressed in MG fruits compared with leaves, and a trend of a rapid increase in *SlHDA1* was observed along with the fruit ripening (Fig. 1B), indicating that *SlHDA1* may be related to tomato fruit ripening. In addition, the expression levels of *SlHDA2* and *SlHDA3*, two homologues of *SlHDA1*, were not affected in *SlHDA1* RNAi lines (Fig. 1C,D), suggesting that the RNAi construct targeting *SlHDA1* is specific and does not target other *HDAC* genes.

### Silencing *SlHDA1* accelerates fruit ripening and enhances carotenoid accumulation

**Figure 2:**
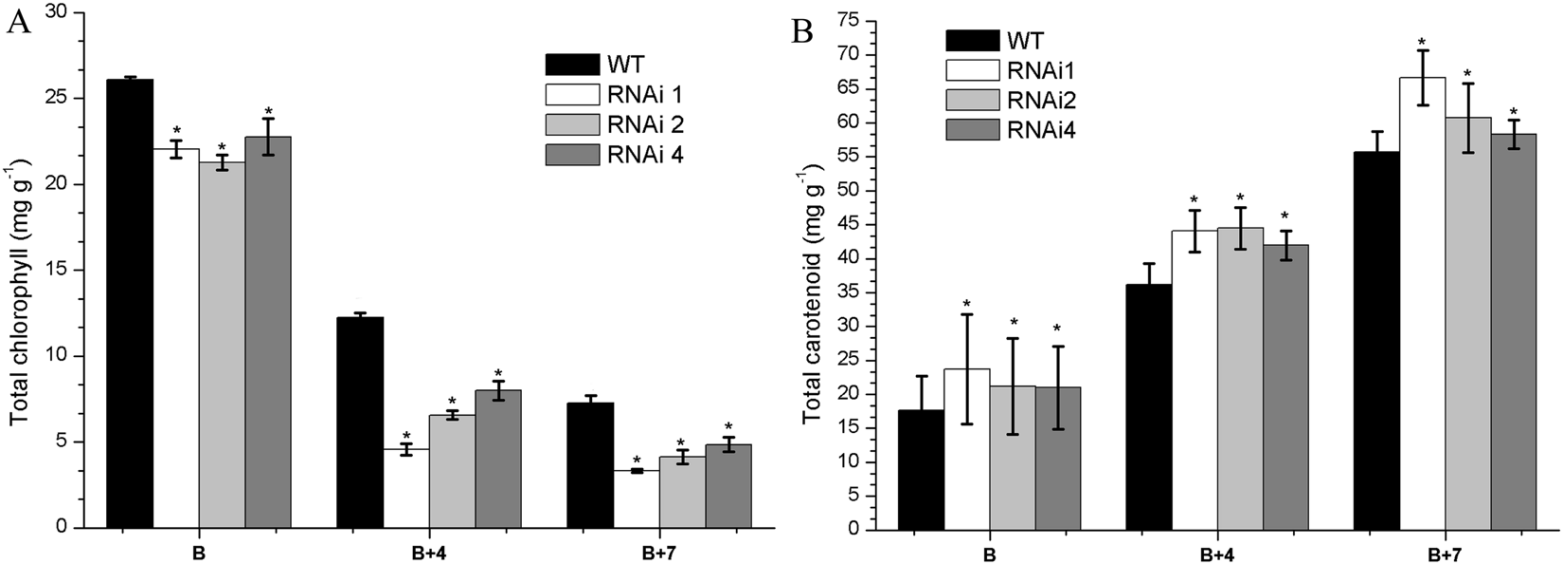
Chl (**A**) and carotenoid (**B**) accumulation profiles between wild type (WT) and *SlHDA1* RNAi fruits in pericarp. B, breaker; B + 4, 4 d after breaker stage; B + 7, 7 d after breaker stage. Biological replicates (3–4 fruits per fruit ripening stage) were performed in triplicate, and the data are presented as means ± SD. The asterisks indicate statistically significant differences between the WT and transgenic fruits (P < 0.05).

The wild type and transgenic tomato plants were grown under normal conditions. Flowers were tagged at anthesis, and the time from the anthesis to ripening stage was measured for the wild type and transgenic lines. Color changes were observed earlier in *SlHDA1* RNAi fruits compared with wild type (Fig. 1E). The ripening time was reported to be 3–6 days earlier in RNAi lines as compared to WT plants(Table 1). It was reported that the dramatic color change from green to red in tomato fruits is caused by chlorophyll degradation and accumulation of carotenoids^25^, including lycopene (red) and β-carotene (orange)^10, 25^. In this study, total Chl and carotenoids in the RNAi lines and wild type fruits at B, B + 4 and B + 7 stages were extracted and determined. As shown in Fig. 2A, total Chl decreased by approximately 50–60% in transgenic lines compared with wild type fruits at the B + 4 and B + 7 stage, and a 15–20% decrease was observed in RNAi lines of fruits at the B stage. In contrast, the total carotenoids increased by 30% in RNAi fruits compared with wild type fruits (Fig. 2B).

**Table 1:** Days from anthesis to breaker stage for Wild type and *SlHDA1* RNAi lines.

Tomato line	Days
Wild type	36.0 ± 0.50
RNAi 1	30.4 ± 0.43
RNAi 2	31.5 ± 0.47
RNAi 4	32.2 ± 0.42

15–20 fruits were examined for biological replicates per line in Table 1.

**Figure 3:**
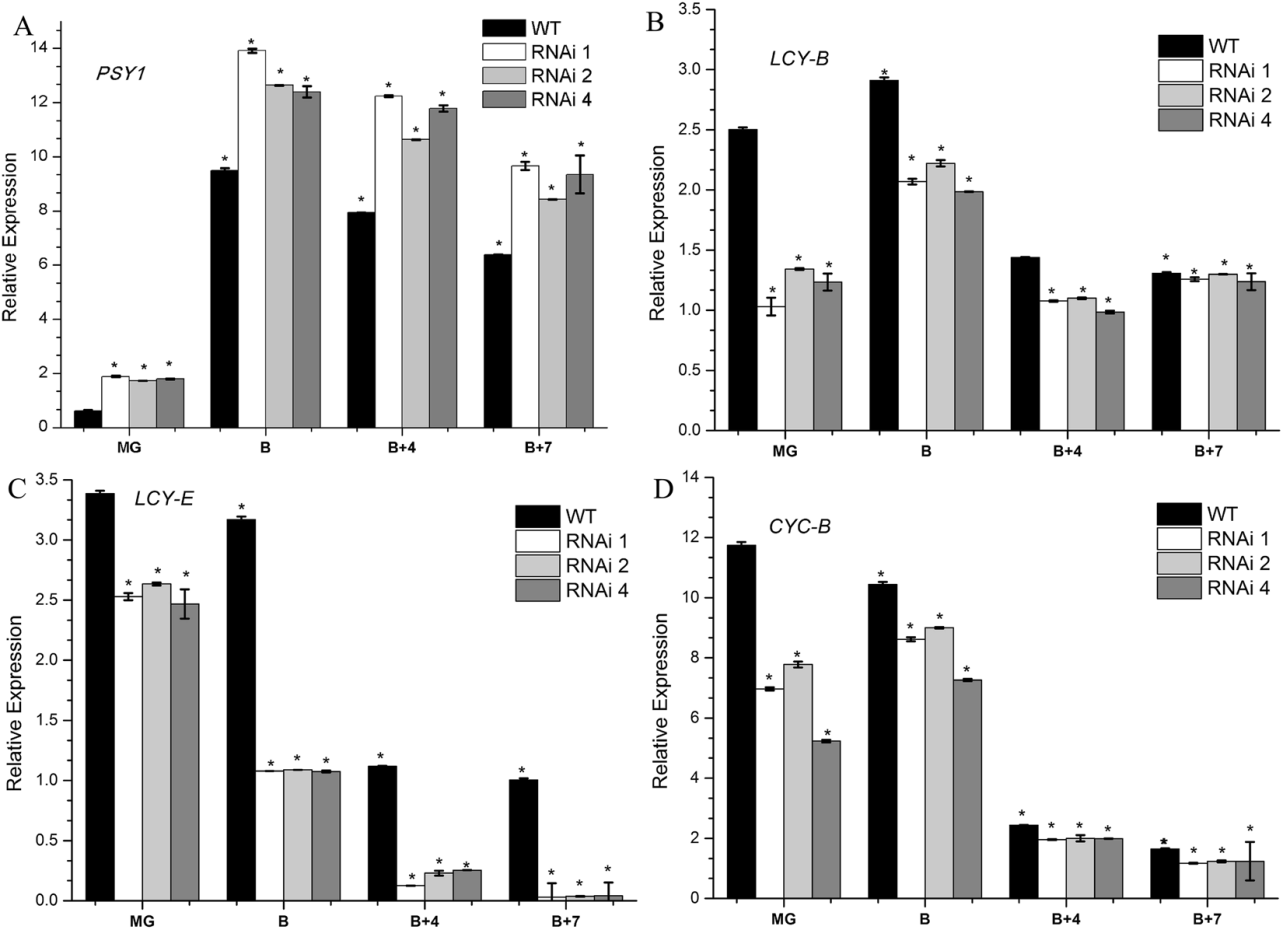
Expression of carotenoid biosynthesis genes *PSY1*, *LCY-B*, *LCY-E* and *CYC-B* in pericarp between wild type (WT) and *SlHDA1* RNAi lines. MG, mature green; B, breaker; B + 4, 4 d after breaker stage; B + 7, 7 d after beaker stage. Three biological replications and three technical replications for each sample were performed. Data are the means ± SD of three independent experiments. The asterisks indicate statistically significant differences between the WT and transgenic fruits (P < 0.05).

To confirm the underlying causes of the differences in color and carotenoid accumulation between the *SlHDA1* RNAi lines and wild type, the expression levels of carotenoid biosynthesis-related genes were measured in the fruit pericarp of *SlHDA1* RNAi lines and wild type from MG to B + 7 stages by quantitative RT-PCR (Fig. 3). The results displayed that *PSY1* (Phytone synthease1) was up-regulated in RNAi fruits, while the expression of *CYC-B*, *LCY-B* and *LCY-E* was remarkably down-regulated in RNAi fruits compared with wild type. These results indicate that silencing *SlHDA1* affects fruit ripening in tomato.

### Reduced expression of *SlHDA1* stimulates ethylene production and ethylene-related gene expression during ripening

**Figure 4:**
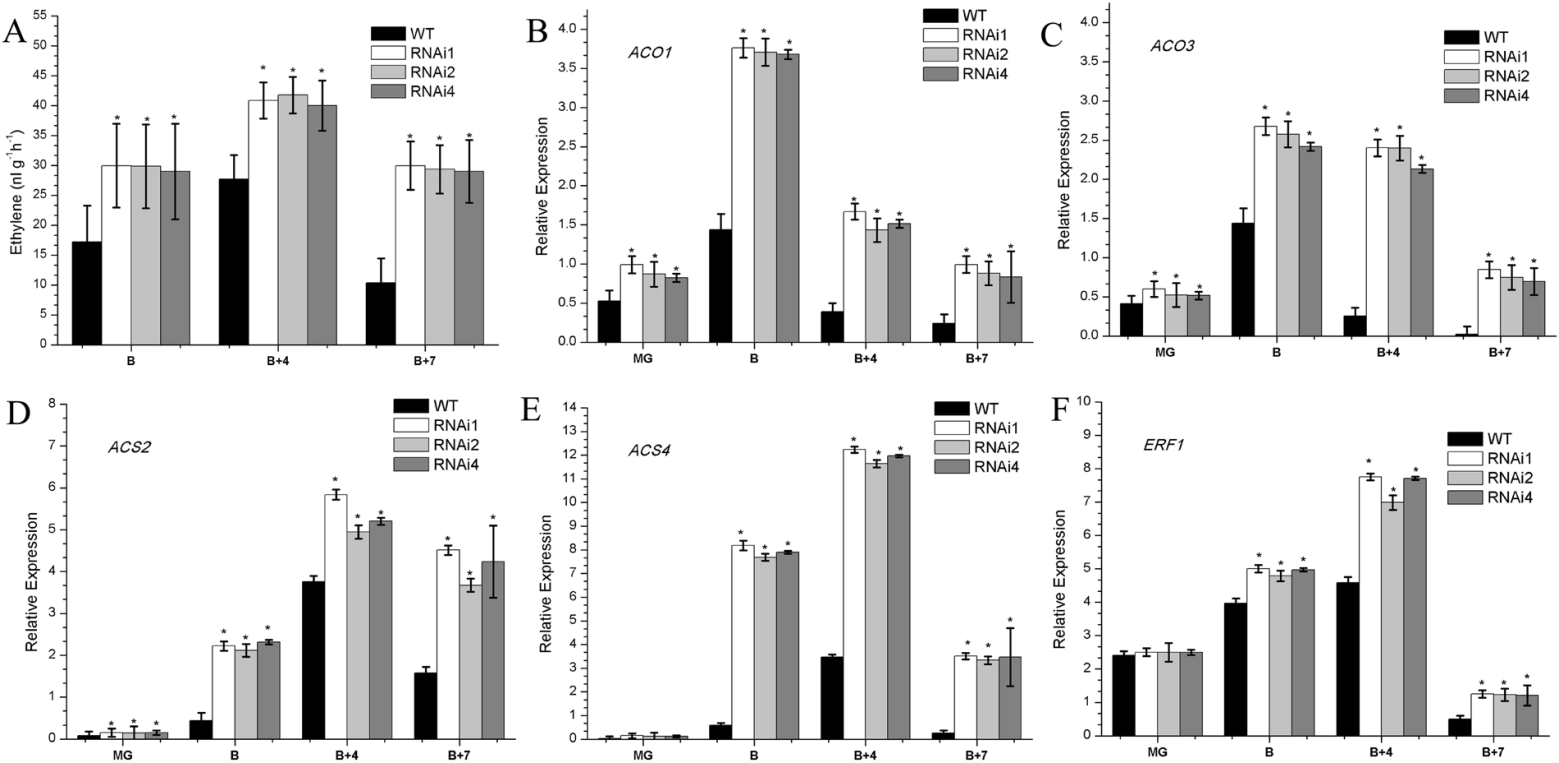
(**A**) Ethylene production and (**B**) to (**F**) relative expression profiles of ethylene biosynthesis related genes *ACO1*, *ACO3*, *ACS2* and *ACS4* and the ethylene response factor *ERF1* in the pericarp between wild type (WT) and *SlHDA1* RNAi fruits. Ethylene production of WT and transgenic fruits was detected at the indicated stage (**B**, B + 4 and B + 7). Three biological replications and three technical replications for each sample were performed. Data are the means ± SD of at least three individual fruits. MG, mature green; B, breaker; B + 4, 4 d after breaker stage; B + 7, 7 d after breaker stage. Gene relative expression data are the means ± SD of three independent experiments. The asterisks indicate statistically significant differences between the WT and transgenic fruits (P < 0.05).

Ethylene biosynthesis, perception and signal transduction are essential for the initiation and completion of tomato fruit ripening^8^. Carotenoid biosynthesis is also regulated by ethylene. To further investigate the relationship between *SlHDA1* and ethylene^26^, ethylene production in *SlHDA1* RNAi fruits and wild type was measured from stages B to B + 7. As shown in Fig. 4A, ethylene production was strongly stimulated in *SlHDA1* RNAi fruits.

Furthermore, the transcript levels of ethylene biosynthesis genes(*ACO1*, *ACO3*, *ACS2* and *ACS4*) and ethylene response factor *ERF1* ^27^ were dramatically up-regulated in RNAi fruit pericarp from B to B + 7 stages (Fig. 4B–F).

### Suppressed expression of *SlHDA1* increases ethylene sensitivity in tomato seedlings

**Figure 5:**
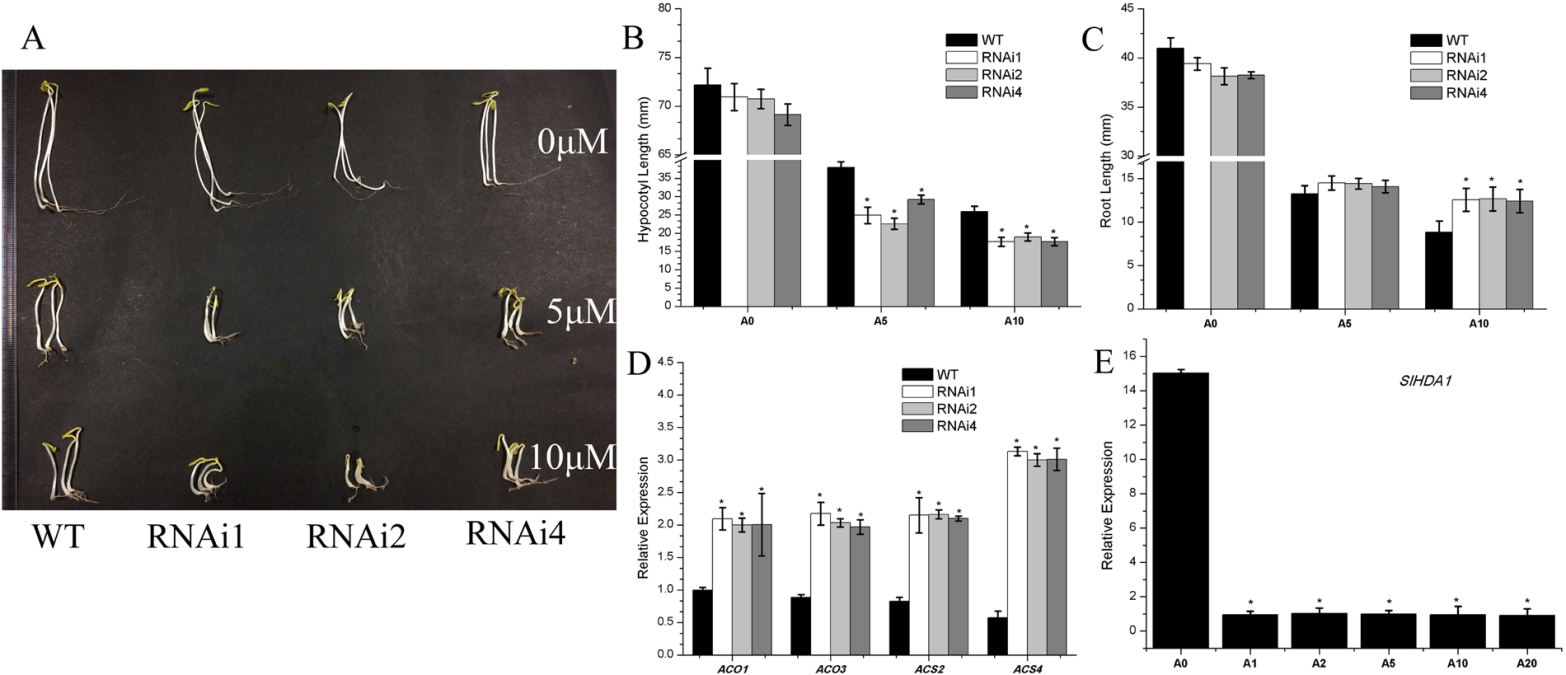
Ethylene triple response assay. (**A**) 20–30 seedlings of wild type (WT) and RNAi lines (RNAi 1, RNAi 2 and RNAi 4) treated with 0, 5.0 and 10.0 µM ACC. (**B**) and (**C**) Elongation of hypocotyls (**B**) and roots (**C**) growth on different concentrations of ACC. (**D**) Expression of *ACS2*, *ACS4* and *ACO1*, *ACO3* in seedlings of RNAi lines and the wild type (WT). (**E**) Expression of *SlHDA1* in seedlings of the wild type treated with 0 (A0), 1.0 (A1), 2.0 (A2), 5.0 (A5), 10.0 (A10), and 20.0 (A20) µM ACC. Three biological replications and three technical replications for each sample were performed. Data are the means ± SD of three independent experiments. The asterisks indicate statistically significant differences between the WT and transgenic fruits (P < 0.05).

To measure the ethylene sensitivity of *SlHDA1* RNAi plants, the ethylene triple response assay was performed. Wild type and *SlHDA1* RNAi seeds were germinated in Murashige and Skoog (MS) medium supplemented with or without the ethylene precursor ACC, which can be taken up by the roots and rapidly converted to ethylene. The elongation of hypocotyls and roots was evaluated 7 days after sowing. The results showed that the average lengths of hypocotyl elongation in RNAi lines were slightly shorter than that of wild type in the absence (0 μM) of ACC, but there were significantly shorter in the presence of ACC (5.0 μM and 10.0 μM) (Fig. 5A and B). In addition, the root elongation of wild type and RNAi lines was nearly identical in the absence (0 μM) of ACC,but RNAi seedlings had longer roots than wild type at higher levels of ACC (5.0 μM and 10.0 μM) (Fig. 5A and C).

Subsequently, the expression of ethylene-related genes was also detected in RNAi lines and wild type seedlings. The results demonstrated that *ACS2*, *ACS4*, *ACO1*, and *ACO3* were all up-regulated significantly in RNAi seedlings in the presence of ACC(5.0 μM) (Fig. 5D). In addition, the transcript of *SlHDA1* in wild type seedlings decreased dramatically after ACC treatment (Fig. 5E), which suggested that *SlHDA1* expression might be impacted by ACC or ethylene.

### Ripening-related genes are significantly up-regulated in *SlHDA1* RNAi fruits

**Figure 6:**
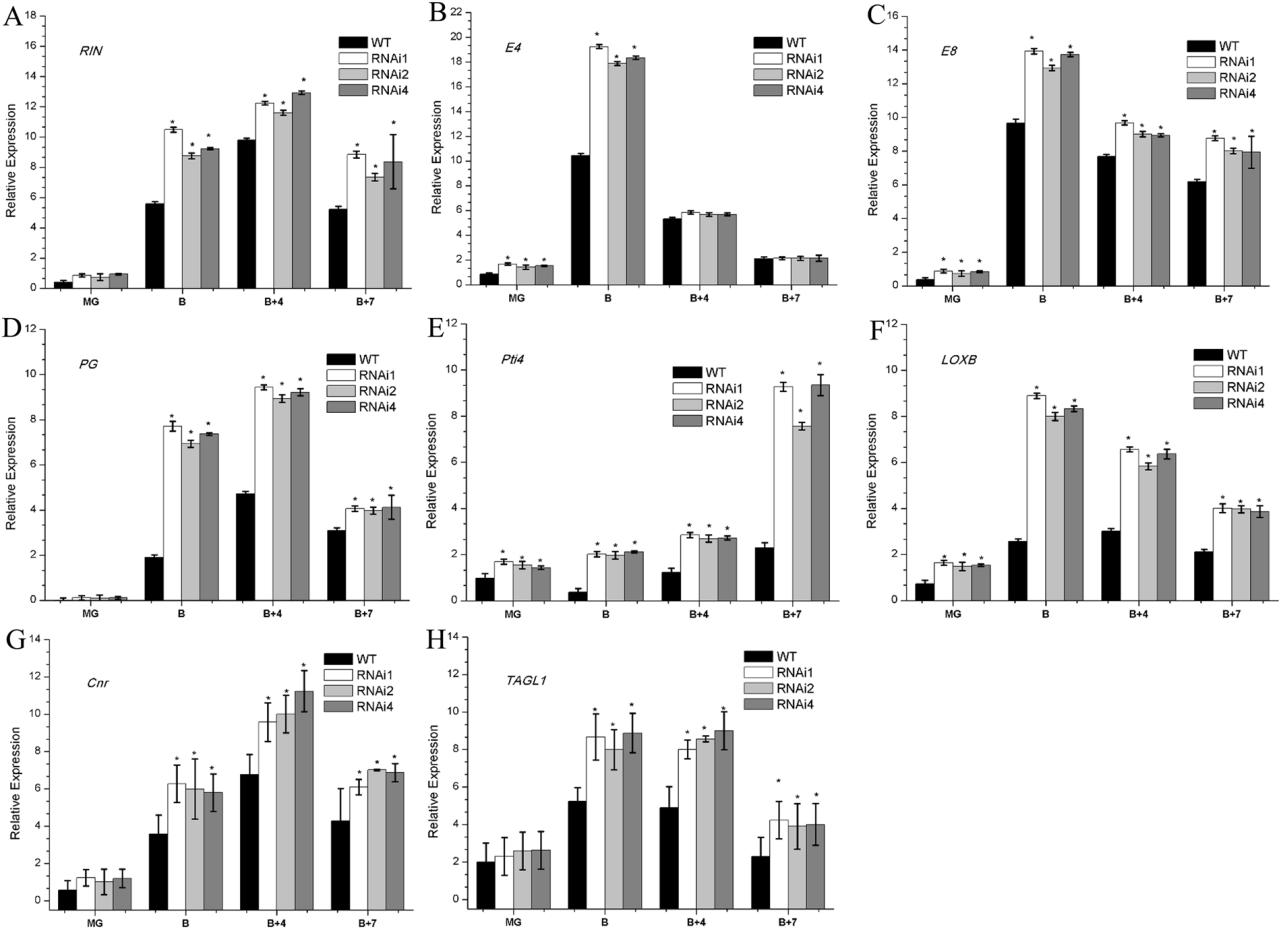
Ripening-associated gene expression profiles in pericarp between wild type (WT) and *SlHDA1* RNAi fruits. MG, mature green; B, breaker; B + 4, 4 d after breaker stage; B + 7, 7 d after beaker stage. Three biological replications and three technical replications for each sample were performed. Data are the means ± SD of three independent experiments. The asterisks indicate statistically significant differences between the WT and transgenic fruits (P < 0.05).

To further characterize the molecular regulation mechanism of *SlHDA1* in fruit ripening, a set of ripening-related genes in wild type and transgenic tomato fruits were examined. Figure 6A–C and G,H show that expression of *RIN, E4, E8, Cnr* and *TAGL1* was markedly increased in the RNAi fruits. Additionally, *LOXB*, a fruit-specific lipoxygenase gene that is induced by ethylene^28^; *PG*, a ripening-related cell wall metabolism gene^29^; and *Pti4*, which is associated with defence responses; were also analyzed. Dramatic increases in the levels of these genes were also observed in transgenic fruits (Fig. 6E,F). These results suggested that silencing *SlHDA1* induces the expression of these ripening-associated genes, subsequently accelerating fruit ripening.

**Figure 7:**
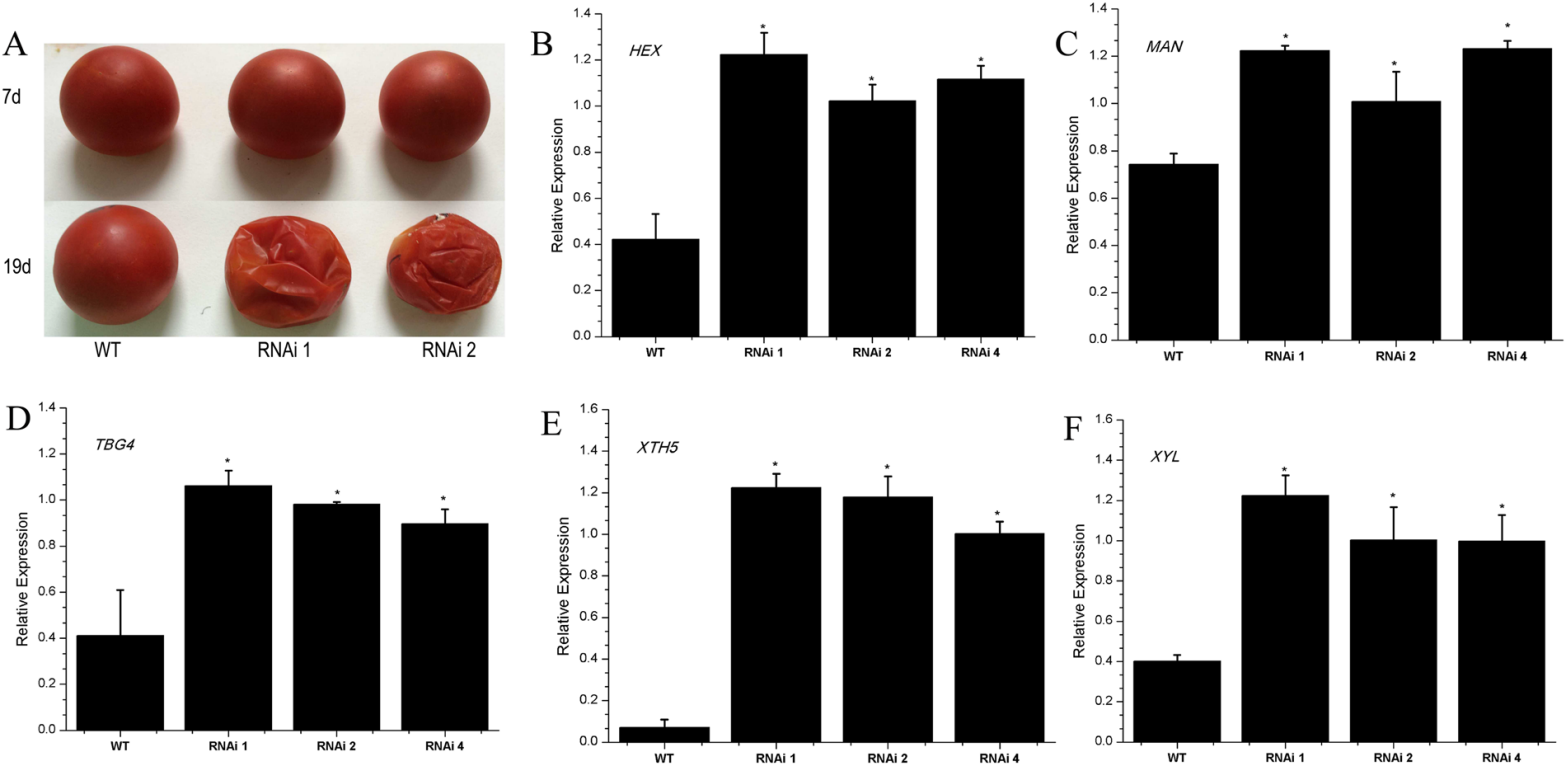
Phenotype and related genes expression of wild type and *SlHDA1* RNAi fruits. (**A**) Fruits storability phenotype of wild type and transgenic lines. 7 d and 19 d, post-harvest storage time. (**B**) to (**F**) Relative expression profiles of related genes in the pericarp between wild type (WT) and *SlHDA1* RNAi fruits. Three biological replications and three technical replications for each sample were performed. Data are the means ± SD of three independent experiments. The asterisks indicate statistically significant differences between the WT and transgenic fruits (P < 0.05).

### *SlHDA1* RNAi fruits have a shorter shelf life

Fruits of wild type and transgenic lines were harvested at the B + 7 stage and stored under the same conditions. Twelve days after harvesting, transgenic tomatoes began to soften, yet wild type fruits remained hard. Nineteen days after being harvested, transgenic tomatoes were soft, dehydrated and moldy, while wild type fruits had just began to soften (Fig. 7A).

To further investigate the molecular mechanism underlying the shorter shelf life of *SlHDA1* RNAi fruit**s**, we measured ripening-related cell wall metabolism genes in the B + 4 stage. As shown in Fig. 7B–F, the transcripts of cell wall metabolism genes, *HEX*, *MAN*, *TBG4*, *XTH* and *XYL* were increased significantly in *SlHDA1* RNAi fruits.

## Discussion

In this study, *SlHDA1*, a histone deacetylase gene was studied by analyzing the phenotype, gene expression and metabolites of *SlHDA1* RNAi fruits. The results indicated that *SlHDA1* is involved in the regulation of tomato carotenoid accumulation and plays a negative role in the fruit ripening regulatory network.

### *SlHDA1* influences carotenoid accumulation during tomato fruit ripening

The carotenoid pigment lycopene is responsible for the red colour of tomato fruits, and its concentration increases dramatically during the ripening process^10, 25^. To date, the biosynthesis of carotenoids has been studied extensively using ripening-deficient mutant fruits^30^. In this pathway, *PSY1* is a major regulator of metabolic flux towards downstream carotenoids^25, 31^. A mutation in *PSY1* causes a yellow-fresh phenotype and an absence of carotenoids in ripe fruit^32, 33^. Furthermore, the cyclization of lycopene is an important branching point in the pathway: one route leads to the production of β-carotene and its derivative xanthophylls (catalyze by LCY-B and CYC-B), whereas the other leads to α-carotene and lutein production (catalyze by LCY-B and LCY-E)^34^. The relative ratio of lycopene and β-carotene in ripening tomato fruit is mediated by up-regulation of *PSY1* and down-regulation of *CYC-B*, which are both regulated by ethylene^25, 35, 36^. In this study, *PSY1* was notably increased in the pericarp of *SlHDA1* RNAi fruits (Fig. 3A), which leads to higher total carotenoid synthesis (Fig. 2B). In contrast, expression of *CYC-B, LCY-B* and *LCY-E* in RNAi fruits was down-regulated compared with wild type (Fig. 3B–D), which alters the carotenoid pathway flux towards lycopene accumulation and away from β-carotene, α-carotene and lutein, thus conferring the darker red fruit phenotype (Fig. 1E). Inversely, NAC4, a plant-specific NAC transcription factor positively regulates carotenoid synthesis, and repression of *NAC4* reduces total carotenoid content and promotes a shift towards β-carotene accumulation in ripening fruits^37^. Together with previous data, our results indicate that *SlHDA1* acts as a negative factor in the regulation of carotenoid biosynthesis and affects lycopene accumulation during tomato fruit ripening.

### *SlHDA1* as an inhibitor influences ethylene biosynthesis and fruit ripening

Ripening of tomato fruits is characterized by an autocatalytic increase in respiration and ethylene biosynthesis just prior to the initiation of ripening. Two modes of ethylene synthesis, system 1 and system 2, are well defined in higher plants^38–40^. System 1 is essential for normal vegetative growth and is responsible for providing the basal level of ethylene that is detectable in all tissues. System 2 produces a large amount of ethylene at the onset of fruit ripening. The ethylene synthesis pathway is well established in higher plants. The key rate-limiting enzymes (ACS and ACO) in ethylene biosynthesis have been cloned and characterized in many species.

In this study, we examined the transcript levels of *ACS2, ACS4*, *ACO1*, and *ACO3* in wild type and *SlHDA1* RNAi fruits and seedlings. The results showed that the transcript levels of *ACS2, ACS4*, *ACO1* and *ACO3* were noticeably higher in RNAi lines than wild type(Figs 4B–E and 5D), suggesting that suppression of *SlHDA1* promotes expression of ethylene biosynthesis genes, which subsequently elevates ethylene production in tomatoes. This was confirmed by measuring the ethylene levels in RNAi fruits. Additionally, root elongation and hypocotyl elongation were slightly shorter in RNAi lines than in the wild type in the absence of ACC, and the triple response assay demonstrated that the RNAi seedlings were more sensitive to ACC than were wild type seedlings (Fig. 5A and C), indicating that more ethylene is probably produced in the RNAi transgenic seedlings. Based on these results, we can speculate that *SlHDA1* impacts ethylene biosynthesis in both vegetative organs and fruits.

Along with fruit ripening, we observed a rapid increase in the transcripts of many ripening-related genes, such as *E4*, *E8*, *PG*, *RIN*, *Pti4*, *Cnr*, *TAGL1* and *LOXB*, *HEX*, *MAN*, *TBG4*, *XTH5* and *XYL*. These genes reflect a range of downstream fruit ripening activities, impacting, for example, carotenoid accumulation, cell wall structure and the production of metabolites associated with softening, flavor, aroma and nutrition^8, 41, 42^. In *SlHDA1* RNAi fruits, the expression of these genes was remarkably upregulated (Figs 6A–H and 7B–F), indicating that suppressing the expression of *SlHDA1* promotes the expression of ripening-related genes and accelerates the rate of ripening and softening. This was supported by the shorter shelf life of RNAi fruits. These results strongly suggest that *SlHDA1* acts as an inhibitor in fruit ripening.

In summary, *SlHDA1* plays a key role in fruit ripening as a negative regulator by modulating carotenoid pigmentation and the climacteric ripening hormone ethylene. Although failure in the detection of acetylation and methylation levels and higher levels of a developmental regulatory cascade of this gene remain to be discovered, as a repressive regulator, *SlHDA1* plays an important role in balancing the activities of positive ripening regulators and adds a new component to the increasingly characterized mechanisms that regulate fleshy fruit ripening. However, whether this mechanism occurs similarly during the ripening of all fleshy fruit species requires further investigation.

## Materials and Methods

### Plant materials and growth conditions

In this study, wild type tomato (*Solanum lycopersicum* Mill. cv. Ailsa Craig) and transgenic plants were planted in a greenhouse under sodium lights for 16 h days (25 °C), 8 h nights (18 °C) and watered daily. Flowers were labeled at anthesis and fruit development was recorded as days post-anthesis (DPA). The ripening stages of tomato fruits were divided according to DPA and fruit color. The ripening stages of wild type tomato fruits were divided into IMG (immature green; 20 DPA), MG (mature green; 33 DPA, full size fruits expansion but no obvious color change), B (breaker; 36DPA, the fruits color changes from green to yellow), B + 4 (4 days after breaker) and B + 7 (7 days after breaker). For all plant samples, total RNA was prepared at the same time each day, and was immediately frozen with liquid nitrogen and stored at −80 °C until required.

### Cloning of *SlHDA1*

Total RNA was isolated from all plant tissues including root, stem, leaf (young leaf, mature leaf and senescent leaf), flower, sepal and fruits (immature green, mature green, B, B + 4 and B + 7) of wild type tomato using Trizol (Invitrogen, USA) according to the manufacturer’s instructions. Then, 2 µg total RNA was used to synthesis first-strand cDNA using the reverse transcription polymerase chain reaction (M-MLV reverse transcriptase, Takara) with an Oligo(dT)_18_ primer. A 1–2 µL sample of cDNA was used to clone the full length *SlHDA1* gene with primers FHDA1-F and FHDA1-R (Supplementary Table S1) using high fidelity PCR (Prime STARTM HS DNA polymerase, Takara). Positive clones were picked out via *Escherichia coli* JM109 transformation and confirmed by sequencing (Invitrogen).

### Construction of the *SlHDA1* RNAi vector and plant transformation

To further study the function of the *SlHDA1* gene, an RNAi vector was constructed. A 309-bp specific DNA fragment of *SlHDA1* was amplified with primers SlHDA1-RNAi-F and SlHDA1-RNAi-R (Supplementary Table S1), which had been tailed with *Hind*III/*Kpn*I and *Xho*I/*Xba*I restriction sites at the 5′end, respectively. Then, the amplified products were digested and linked into the pHANNIBAL plasmid at the *Hind*III/*Kpn*I restriction site in the sense orientation and at the *Xho*I/*Xba*I restriction site in the antisense orientation. Finally, the double-stranded RNA expression unit, which includes the cauliflower mosaic virus 35 S promoter, the *SlHDA1* fragment in the antisense orientation, a PDK intron, the *SlHDA1* fragment in the sense orientation, and the OCS terminator, was purified and inserted into the plant binary vector pBIN19 using *Sac*I and *Xba*I restriction sites. The resulting construct was transformed into tomato cv. Ailsa Craig using *Agrobacterium tumefaciens* (strain LBA4404) by the freeze-thaw method. Transformed lines were selected for kanamycin (80 mg l^−1^) resistance and then analyzed by PCR to determine the presence of T-DNA using the primers NPTII-F/R (Supplementary Table S1). The positive transgenic plants were selected and used for subsequent experiments.

### Quantitative RT–PCR analysis

The RNA extraction from all plant tissues including root, stem, leaf (young leaf, mature leaf and senescent leaf), flower, sepal and fruits (immature green, mature green, B, B + 4 and B + 7) of wild type and homozygous T2 transgenic plants, and cDNA synthesis were performed as described earlier. The synthesized cDNAs were diluted 2 times with RNase/DNase-free water. Quantitative real-time PCR analysis was performed using the CFX96^TM^ Real-Time System (C1000^TM^ Thermal Cycler, Bio-Rad). All reactions were carried out using the SYBR^®^ Premix Go Taq II kit (Promega, China) in a 10 µL total sample volume (5.0 µL of 2 × SYBR Premix Go Taq, 0.5 µL of primers, 1.0 µL of cDNA, 3.5 µL of ddH_2_O). For analysis of each gene, an NRT (no reverse transcription control) and NTC (no template control) were also performed. The tomato *SlCAC* gene and *SlEF1a* gene were also evaluated to be used as the internal standards for development studies^43^ and abiotic stress studies, respectively^44^. The relative gene expression levels were conducted using the 2^−△△C^_T_ method^45^. Primers used for quantitative RT-PCR are shown in Supplementary Table S2. Three biological replicates and three technical replicates were used for RT-PCR analyses, respectively.

### Ethylene measurements

Fruits from the B, B + 4 and B + 7 stages were harvested and placed in open 100 mL jars for 3 h to minimize the effects of wound induced ethylene production caused by picking. Jars were then sealed and incubated at room temperate for 24 h and 1 mL of headspace gas was injected into a Hewlett-Packard 5890 series gas chromatograph equipped with a flame ionization detector. Samples were compared with standards of known concentration and normalized for fruit weight^46^. Three biological replicates and three technical replicates were used for ethylene measurements.

### Pigment quantification in tomato fruit

Tomato pigments were extracted from pericarp using a modified protocol from the previous report^47^. 1.0 g sample were cut from pericarp in a 5 mm wide strip around the equator of MG, B, B + 4 and B + 7 of wild type and RNAi lines, respectively. Then grounded them with liquid nitrogen and 20 ml of 60: 40% (v/v) hexane: acetone. The extract was centrifuged at 4000 × g for 5 min and the supernatant was carefully transferred to a new tube. The sediment were repeatedly extracted with fresh solvent until colorless and the absorbance of supernatant was measured at 450 nm, 647 nm and 663 nm, respectively. The total Chl and carotenoid contents were calculated with the following equations: total Chl mg ml^−1^ = 8.02(OD_663_) + 20.2(OD_647_) and total carotenoids mg ml^−1^ = (OD_450_)/0.25. Individual tissue samples were taken from 3–4 fruits for each ripening stage in biological triplicate and three times for technical replicates.

### Postharvest storage test

Fruits of wild type and RNAi lines were harvested at B stage, and placed on filter paper in greenhouse conditions. Phenotype was observed every two days.

### Ethylene triple response assay

The seeds of wild type plants were sterilized and sown on MS medium supplemented with 0, 0.5, 1.0, 2.0, 5.0, 10.0, and 20.0 μM ACC and then cultured in the dark at 25 °C. Meanwhile, T1 seeds of RNAi lines were sterilized and sown on MS medium supplemented with 0, 5.0 and 10.0 μM ACC and then cultured under the same conditions as the wild type plants. Hypocotyl and root elongation were measured 7 days after sowing, and at least 20 seedlings were measured for each culture. To further explore the molecular mechanism of the triple response of transgenic lines, the expression of *ACO1*, *ACO3*, *ACS2* and *ACS4* in the wild type and transgenic lines were measured by qPCR. The expression of *SlHDA1* was also detected in wild type seedlings treated with 0, 1.0, 2.0, 5.0, 10.0, and 20.0 μM ACC.

### Statistical analysis

Data were analyzed by one-way analysis of variance (ANOVA) and different means were significant by a *t-*test at P < 0.05.

## Electronic supplementary material

Supplementary Table S1 and S2
