# Supplementary material for: The tomato histone deacetylase SlHDA1 contributes to the repression of fruit ripening and carotenoid accumulation
Source: Sci Rep. 2017 Aug 11;7:7930. doi: 10.1038/s41598-017-08512-x (PMC5554242; doi:10.1038/s41598-017-08512-x)
Supplement: Supplementary file 1 — Supplementary Table S1 and S2 [file 41598_2017_8512_MOESM1_ESM.pdf]

Title page:

Title:

The tomato histone deacetylase *SlHDA1* contributes to the repression of fruit ripening and carotenoid accumulation

Authors

Jun-E Guo, Zongli Hu, Mingku Zhu, Fenfen Li, Zhiguo Zhu, Yu Lu, Guoping Chen*

Laboratory of molecular biology of tomato (Chongqing University), Ministry of Education, Bioengineering College, Chongqing University, Chongqing 400044, People's Republic of China

First author: Jun-E Guo

Corresponding author: Guoping Chen

Postal Address: Room 523, Bioengineering College, Chongqing University, Campus B, 174 Shapingba Main Street, Chongqing 400030, P.R. China;

Tel: 00862365112674;

Fax: 00862365112674;

E-mail: chenguoping@cqu.edu.cn

* Corresponding author. Tel:00862365112674; Fax: 00862365112674; E-mail: chenguoping@cqu.edu.cn.

Supplementary Table S1. Specific primer sequences used for gene amplification and cloning procedures.

Primer code	Primer sequences (5' →3')	Application
FHDA1-F	TTATCCTTCTGGTTTATAGCTTGG	Full-length amplification for *SlHDA1*
FHDA1-R	GGTGAGTGAGGGAGATTGT	
SlHDA1-RNAi-F	CGGGGTACCGGATCCCTGAAGGAAAAGGCACGG	To establish *SlHDA1* RNAi lines; added *Kpn* I and *Hin*d III site underlined
SlHDA1-RNAi-R	CCGCTCGAGTCTAGAGAGGGAGATTGTTCATGGATC	To establish *SlHDA1* RNAi lines; added *Xho* I and *Xba* I site underlined
NPTII-F	GACAATCGGCTGCTCTGA	Positive transgenic plants detection
NPTII-R	AACTCCAGCATGAGATCC	

Supplementary Table S2. Specific primer sequences used for qRT-PCR analysis.

Primer code	Primer sequences (5' →3')	Application
CAC-Q-F	CCTCCGTTGTGATGTAACTGG	Internal standard gene for Quantitative RT-PCR in tomato development
CAC-Q-R	ATTGGTGGAAAGTAACATCATCG	
EF1α-Q-F	TACTGGTGGTTTTGAAGCTG	Internal standard gene for Quantitative RT-PCR under abiotic stress
EF1α-Q-R	AACTTCCTTCACGATTTCATCATA	
SlHDA1-Q-F	CCTACGCTGGAGGTTCTGTTG	Quantitative RT-PCR analysis for *SlHDA1*
SlHDA1-Q-R	GCTCCAGAATAGCCAACACGA	
PSY1-Q-F	AGAGGTGGTGGAAAGCAA	Quantitative RT-PCR analysis for carotenoid pathway genes
PSY1-Q-R	TCTCGGGAGTCATTAGCAT	
LCYB-Q-F	TTGACTTAGAACCTCGTTATTGG	
LCYB-Q-R	AACAGTTCCCTTTGTCATTATCTC	
LCYE-Q-F	GCCACAGGTTATTCAGTCGTCA	
LCYE-Q-R	CCAGTCCAAATAGGAAAAACGAT	
CYCB-Q-F	CGACGTGATCATTATCGGAGC	
CYCB-Q-R	GTGGTGAAGGGTCAACACAACA	
ACO1-Q-F	ACAAACAGACGGGACACGAA	Quantitative RT-PCR analysis for ethylene-related genes
ACO1-Q-R	CTCTTTGGCTTGAAACTTGA	
ACO3-Q-F	CAAGCAAGTTTATCCGAAAT	
ACO3-Q-R	CATTAGCTTCCATAGCCTTC	
ACS2-Q-F	GAAAGAGTTGTTATGGCTGGTG	
ACS2-Q-R	GCTGGGTAGTATGGTGAAGGT	
ACS4-Q-F	GCTCGGAGGTAGGATGGTTTC	
ACS4-Q-R	GTTCCTCTTCCATTGTGCTTGT	
ERF1-Q-F	TTTTAGTATCGGATGGACG	
ERF1-Q-R	GGCGGAGAAACAGAAGTA	
E4-Q-F	AGGGTAACAACAGCAGTAGCA	Quantitative RT-PCR analysis for ripening-related genes
E4-Q-R	CCCAACCTCCGTCTTCAC	
E8-Q-F	GGCACCATTCAACATACCG	
E8-Q-R	CTTTCACCGAAGAAGCACG	
LOXB-Q-F	TGCTACAATGACTTGGGTGAA	
LOXB-Q-R	CCTGTCCTGCCTCTACG	
RIN-Q-F	GGAACCCAAACTTCATCAGA	
RIN-Q-R	TTGTCCCAAATCCTCACCTA	
PG-Q-F	ATACAACAGTTTTCAGCAGTTCAAGT	
PG-Q-R	GGTTTTCCACTTTCCCCTACTAA	
Cnr-Q-F	CGGCAACTCCTCTTAGCATC	
Cnr-Q-R	GCCACAAGGTGTGTGAGTTC	
TAGL1-Q-F	AAAAGAGGGAGATTGAGCTGC	
TAGL1-Q-R	CTCTACCTCTGCTATCTTTGCG	
Pti4-Q-F	CTCTAAGCGTCGGATGGTC	
Pti4-Q-R	AATGTCTTCCTTTCGGTGTTT	

XTH5-Q-F	CCACCACCAGAGTGCGAGAT	Quantitative RT-PCR analysis for fruit cell wall metabolism-related genes
XTH5-Q-R	TTTTCTTAGGATGACGATGTCCG	
HEX-Q-F	GCAGAAGCATTGTGGTCAGGA	
HEX-Q-R	TCAGCACCTATTCCCCTAGAAAC	
TBG4-Q-F	AAATGGTGAAGGCGTAGGTCG	
TBG4-Q-R	AGGTTGTCCGCAGTTAGTCTGG	
XYL-Q-F	TGATCGGCAATTATGAAGGTATTC	
XYL-Q-R	CAGCACATCCTGGCTTGTAAAT	
MAN-Q-F	ACACCGTCCTCCTGAGATTGG	
MAN-Q-R	GAGCCTCTGCTTTCCACTTTAATC	
